# Supplementary figures and images for: VEFill: a model for accurate and generalizable deep mutational scanning score imputation across protein domains
Source: bioRxiv. 2025 Aug 14:2025.05.14.653991. Originally published 2025 May 14. Preprint. [Version 2] doi: 10.1101/2025.05.14.653991 (PMC12132439; doi:10.1101/2025.05.14.653991)

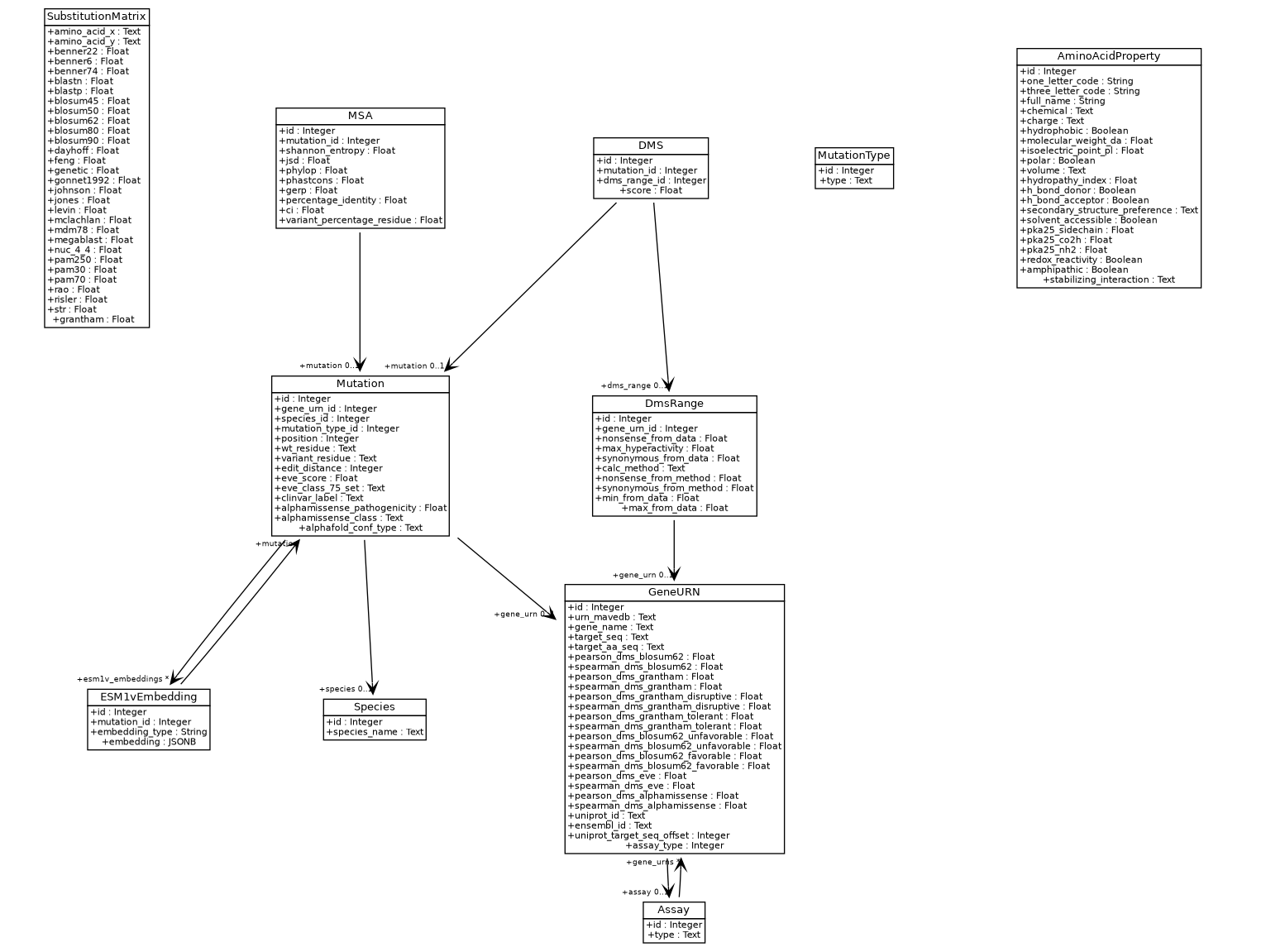

Supplement: Supplement 1 — Supplementary Table S1. Metadata for domains used in the 140-, 521-, and 127-domain (Pfam-unique) model datasets. Includes MaveDB accession, domain name, species, UniProt ID, Pfam ID. Train/test split assignments are indicated for each dataset where applicable. Supplementary Table S2. List of the 25 amino acid substitution matrices used in VEFill. Each row includes the matrix name, its source (Biopython or external repository), the corresponding VEFill feature name, and the original citation. Supplementary Table S3. Complete list of input features used in the VEFill model. Supplementary Table S4. Performance comparison of different model architectures using the same input feature set. Models evaluated include LightGBM (used in VEFill), a fully connected neural network (FCNN), and a transformer-based architecture. Reported metrics include RMSE, MAE, and R2 for both training and test sets. Variants of the FCNN included adjustments to regularization, architecture depth, and optimizer settings. All models were trained using the same 140-domain dataset. Supplementary Table S5. Performance metrics (RMSE, MAE, R2, Pearson r) for both the training and test sets of the general VEFill model, evaluated across multiple feature set configurations. Each row represents a unique feature combination used to evaluate model performance. Supplementary Table S6. Domain-wise evaluation results for the general model under a LOPO strategy. The table reports RMSE, MAE, coefficient of determination (R2), and Pearson correlation (r) for both the training and test sets across 140 individual protein domains. Additionally, the number of mutations in the training and test sets for each domain is provided. Supplementary Table S7. Per-protein model performance metrics (RMSE, MAE, R2, Pearson r) for both training and test sets for 140 domains using an 90/10 random train/test split. Models were trained using all input features except ESM-1v difference vector. The table also includes the number of mut [file media-1.zip › Supplementary_Fig_S1.png]

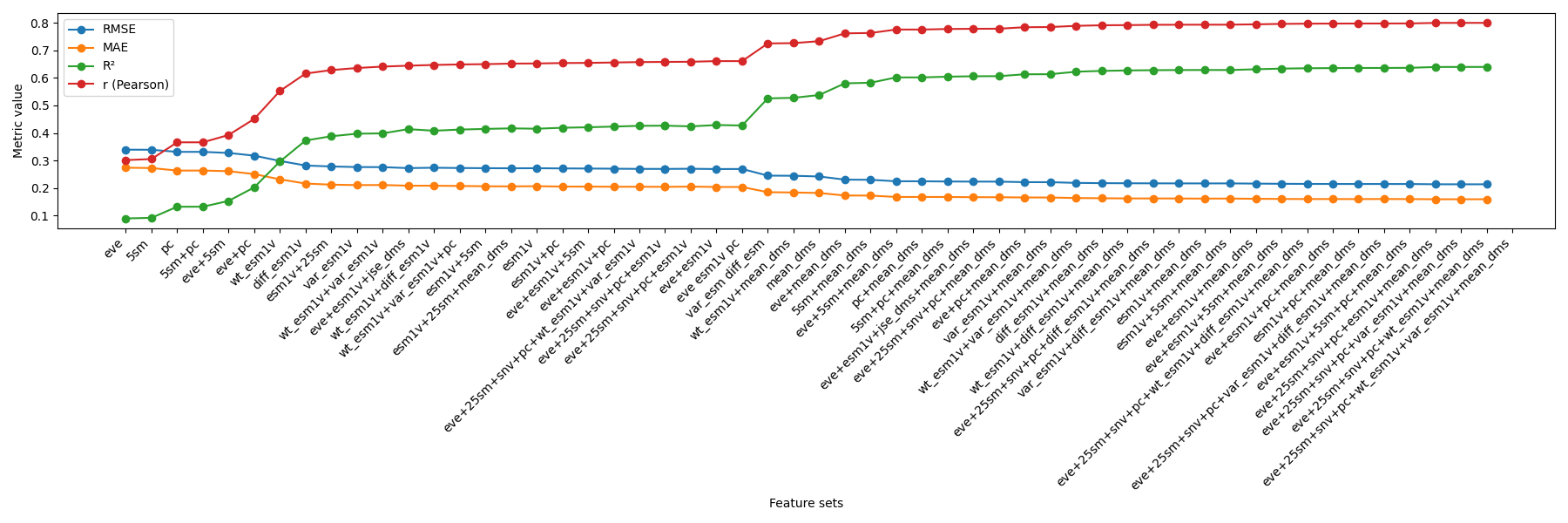

Supplement: Supplement 1 — Supplementary Table S1. Metadata for domains used in the 140-, 521-, and 127-domain (Pfam-unique) model datasets. Includes MaveDB accession, domain name, species, UniProt ID, Pfam ID. Train/test split assignments are indicated for each dataset where applicable. Supplementary Table S2. List of the 25 amino acid substitution matrices used in VEFill. Each row includes the matrix name, its source (Biopython or external repository), the corresponding VEFill feature name, and the original citation. Supplementary Table S3. Complete list of input features used in the VEFill model. Supplementary Table S4. Performance comparison of different model architectures using the same input feature set. Models evaluated include LightGBM (used in VEFill), a fully connected neural network (FCNN), and a transformer-based architecture. Reported metrics include RMSE, MAE, and R2 for both training and test sets. Variants of the FCNN included adjustments to regularization, architecture depth, and optimizer settings. All models were trained using the same 140-domain dataset. Supplementary Table S5. Performance metrics (RMSE, MAE, R2, Pearson r) for both the training and test sets of the general VEFill model, evaluated across multiple feature set configurations. Each row represents a unique feature combination used to evaluate model performance. Supplementary Table S6. Domain-wise evaluation results for the general model under a LOPO strategy. The table reports RMSE, MAE, coefficient of determination (R2), and Pearson correlation (r) for both the training and test sets across 140 individual protein domains. Additionally, the number of mutations in the training and test sets for each domain is provided. Supplementary Table S7. Per-protein model performance metrics (RMSE, MAE, R2, Pearson r) for both training and test sets for 140 domains using an 90/10 random train/test split. Models were trained using all input features except ESM-1v difference vector. The table also includes the number of mut [file media-1.zip › Supplementary_Fig_S2.png]

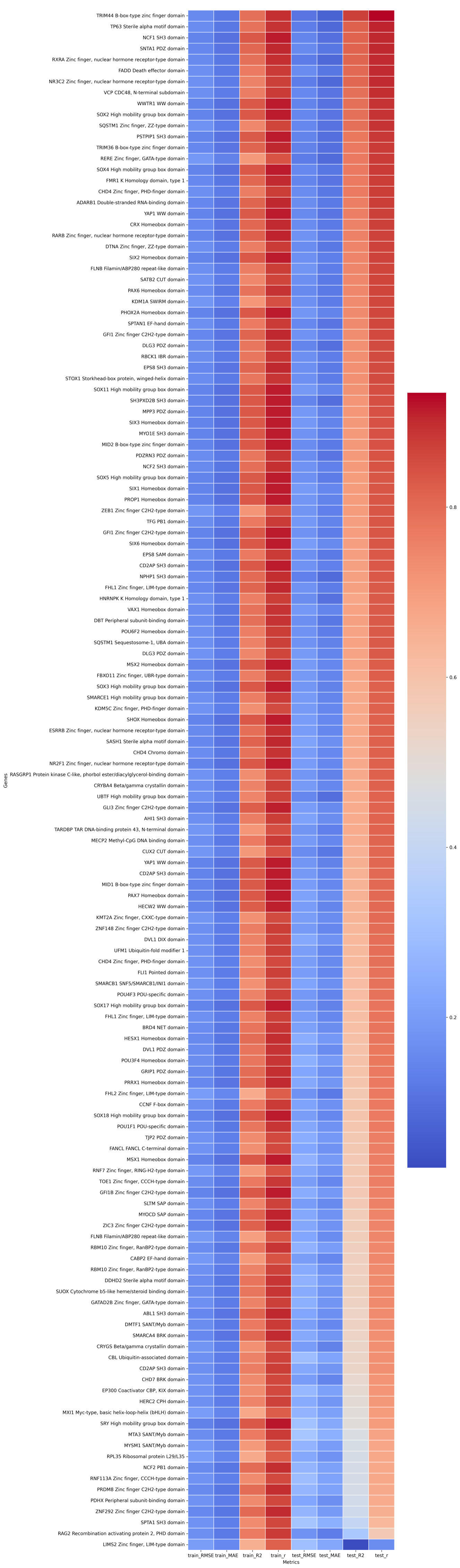

Supplement: Supplement 1 — Supplementary Table S1. Metadata for domains used in the 140-, 521-, and 127-domain (Pfam-unique) model datasets. Includes MaveDB accession, domain name, species, UniProt ID, Pfam ID. Train/test split assignments are indicated for each dataset where applicable. Supplementary Table S2. List of the 25 amino acid substitution matrices used in VEFill. Each row includes the matrix name, its source (Biopython or external repository), the corresponding VEFill feature name, and the original citation. Supplementary Table S3. Complete list of input features used in the VEFill model. Supplementary Table S4. Performance comparison of different model architectures using the same input feature set. Models evaluated include LightGBM (used in VEFill), a fully connected neural network (FCNN), and a transformer-based architecture. Reported metrics include RMSE, MAE, and R2 for both training and test sets. Variants of the FCNN included adjustments to regularization, architecture depth, and optimizer settings. All models were trained using the same 140-domain dataset. Supplementary Table S5. Performance metrics (RMSE, MAE, R2, Pearson r) for both the training and test sets of the general VEFill model, evaluated across multiple feature set configurations. Each row represents a unique feature combination used to evaluate model performance. Supplementary Table S6. Domain-wise evaluation results for the general model under a LOPO strategy. The table reports RMSE, MAE, coefficient of determination (R2), and Pearson correlation (r) for both the training and test sets across 140 individual protein domains. Additionally, the number of mutations in the training and test sets for each domain is provided. Supplementary Table S7. Per-protein model performance metrics (RMSE, MAE, R2, Pearson r) for both training and test sets for 140 domains using an 90/10 random train/test split. Models were trained using all input features except ESM-1v difference vector. The table also includes the number of mut [file media-1.zip › Supplementary_Fig_S3.png]

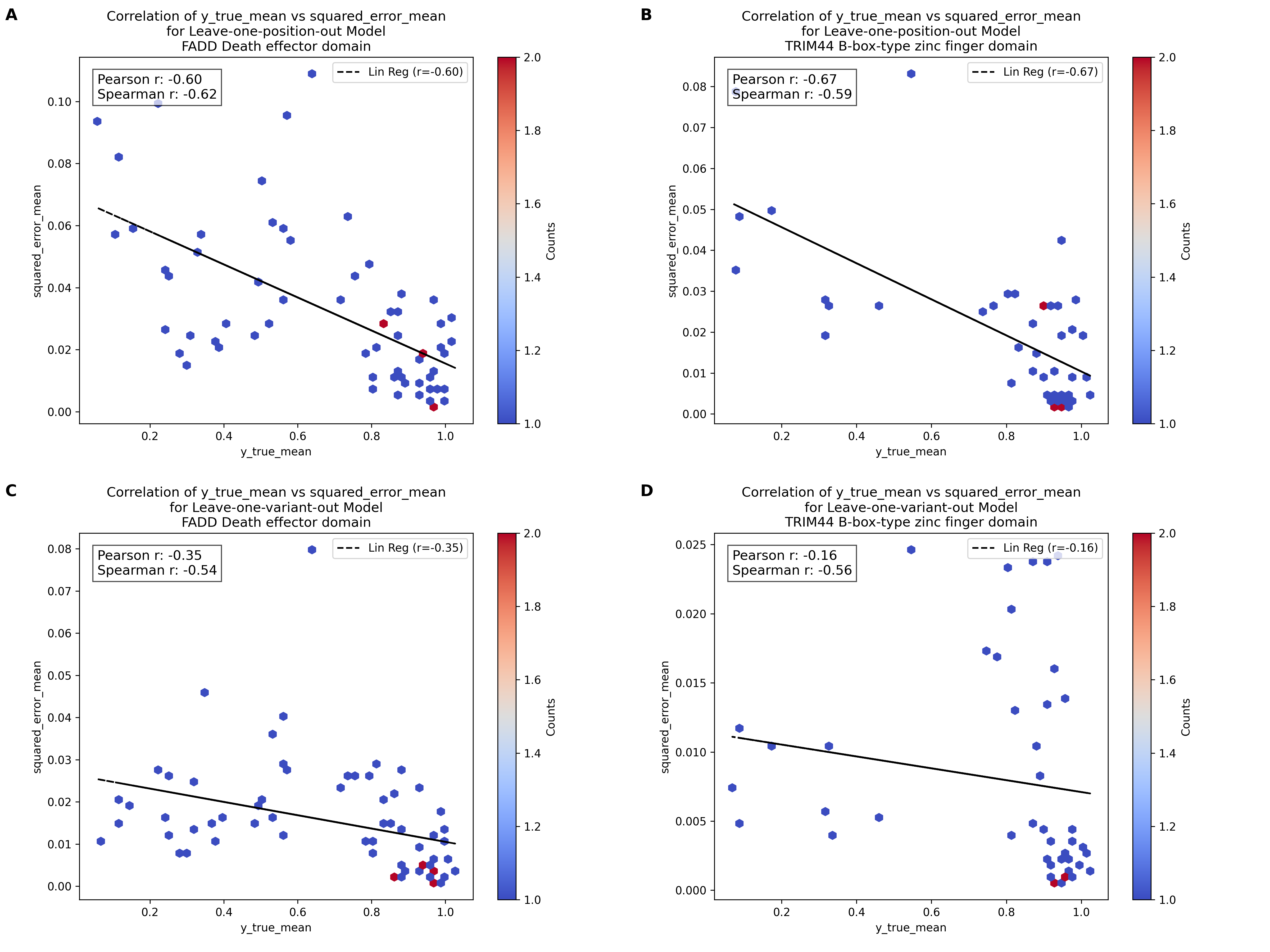

Supplement: Supplement 1 — Supplementary Table S1. Metadata for domains used in the 140-, 521-, and 127-domain (Pfam-unique) model datasets. Includes MaveDB accession, domain name, species, UniProt ID, Pfam ID. Train/test split assignments are indicated for each dataset where applicable. Supplementary Table S2. List of the 25 amino acid substitution matrices used in VEFill. Each row includes the matrix name, its source (Biopython or external repository), the corresponding VEFill feature name, and the original citation. Supplementary Table S3. Complete list of input features used in the VEFill model. Supplementary Table S4. Performance comparison of different model architectures using the same input feature set. Models evaluated include LightGBM (used in VEFill), a fully connected neural network (FCNN), and a transformer-based architecture. Reported metrics include RMSE, MAE, and R2 for both training and test sets. Variants of the FCNN included adjustments to regularization, architecture depth, and optimizer settings. All models were trained using the same 140-domain dataset. Supplementary Table S5. Performance metrics (RMSE, MAE, R2, Pearson r) for both the training and test sets of the general VEFill model, evaluated across multiple feature set configurations. Each row represents a unique feature combination used to evaluate model performance. Supplementary Table S6. Domain-wise evaluation results for the general model under a LOPO strategy. The table reports RMSE, MAE, coefficient of determination (R2), and Pearson correlation (r) for both the training and test sets across 140 individual protein domains. Additionally, the number of mutations in the training and test sets for each domain is provided. Supplementary Table S7. Per-protein model performance metrics (RMSE, MAE, R2, Pearson r) for both training and test sets for 140 domains using an 90/10 random train/test split. Models were trained using all input features except ESM-1v difference vector. The table also includes the number of mut [file media-1.zip › Supplementary_Fig_S5.png]

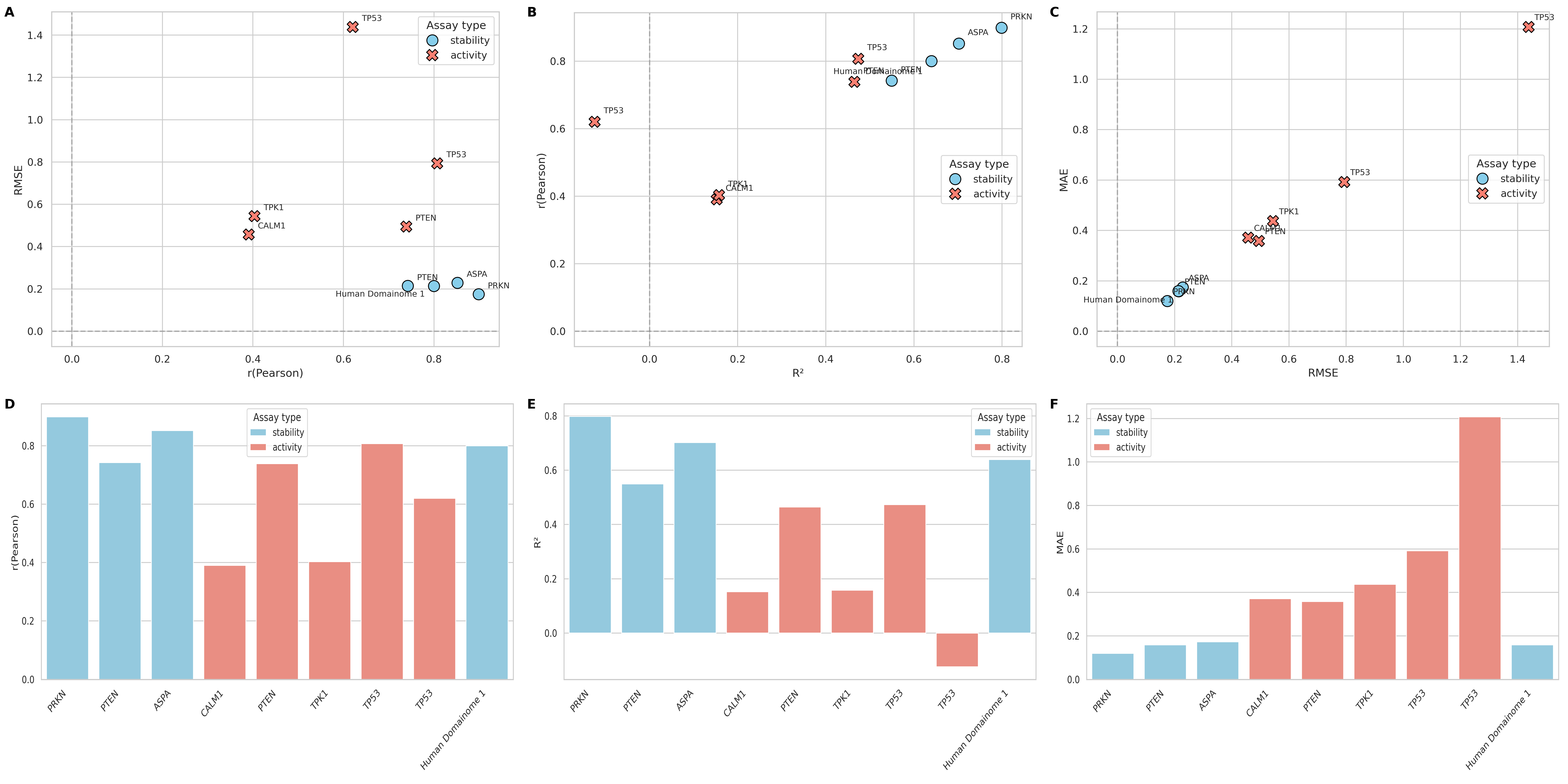

Supplement: Supplement 1 — Supplementary Table S1. Metadata for domains used in the 140-, 521-, and 127-domain (Pfam-unique) model datasets. Includes MaveDB accession, domain name, species, UniProt ID, Pfam ID. Train/test split assignments are indicated for each dataset where applicable. Supplementary Table S2. List of the 25 amino acid substitution matrices used in VEFill. Each row includes the matrix name, its source (Biopython or external repository), the corresponding VEFill feature name, and the original citation. Supplementary Table S3. Complete list of input features used in the VEFill model. Supplementary Table S4. Performance comparison of different model architectures using the same input feature set. Models evaluated include LightGBM (used in VEFill), a fully connected neural network (FCNN), and a transformer-based architecture. Reported metrics include RMSE, MAE, and R2 for both training and test sets. Variants of the FCNN included adjustments to regularization, architecture depth, and optimizer settings. All models were trained using the same 140-domain dataset. Supplementary Table S5. Performance metrics (RMSE, MAE, R2, Pearson r) for both the training and test sets of the general VEFill model, evaluated across multiple feature set configurations. Each row represents a unique feature combination used to evaluate model performance. Supplementary Table S6. Domain-wise evaluation results for the general model under a LOPO strategy. The table reports RMSE, MAE, coefficient of determination (R2), and Pearson correlation (r) for both the training and test sets across 140 individual protein domains. Additionally, the number of mutations in the training and test sets for each domain is provided. Supplementary Table S7. Per-protein model performance metrics (RMSE, MAE, R2, Pearson r) for both training and test sets for 140 domains using an 90/10 random train/test split. Models were trained using all input features except ESM-1v difference vector. The table also includes the number of mut [file media-1.zip › Supplementary_Fig_S6.png]

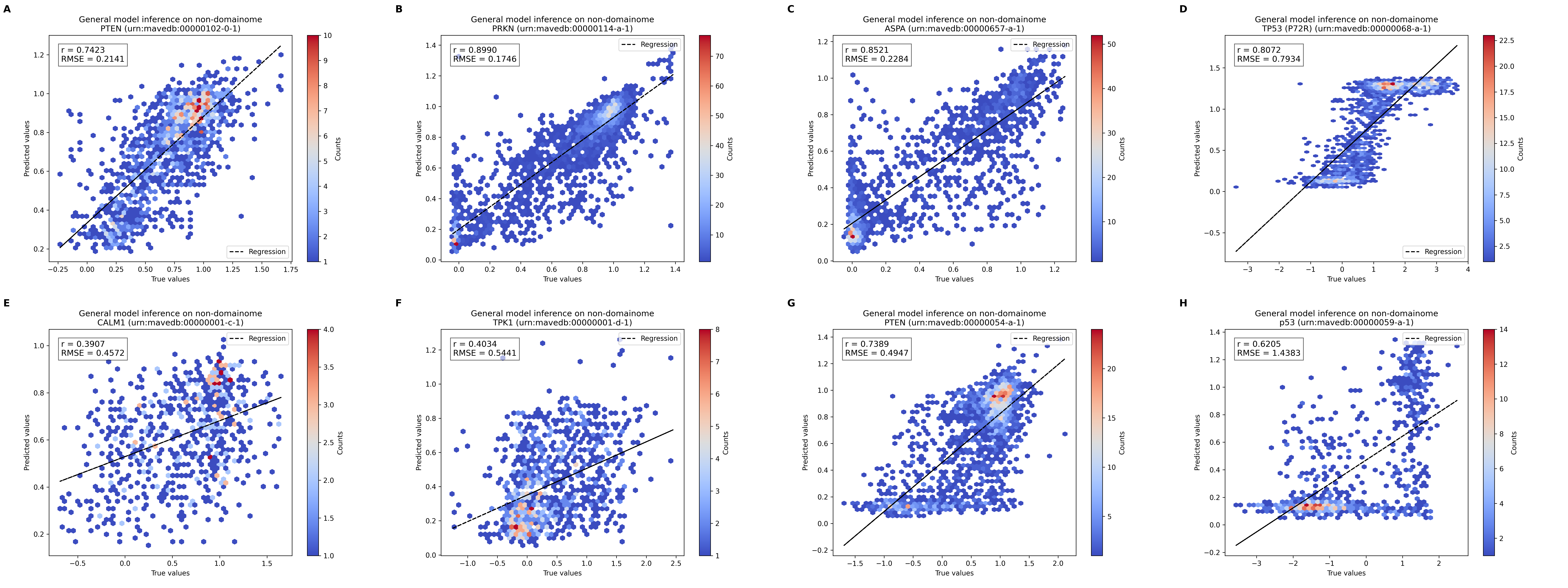

Supplement: Supplement 1 — Supplementary Table S1. Metadata for domains used in the 140-, 521-, and 127-domain (Pfam-unique) model datasets. Includes MaveDB accession, domain name, species, UniProt ID, Pfam ID. Train/test split assignments are indicated for each dataset where applicable. Supplementary Table S2. List of the 25 amino acid substitution matrices used in VEFill. Each row includes the matrix name, its source (Biopython or external repository), the corresponding VEFill feature name, and the original citation. Supplementary Table S3. Complete list of input features used in the VEFill model. Supplementary Table S4. Performance comparison of different model architectures using the same input feature set. Models evaluated include LightGBM (used in VEFill), a fully connected neural network (FCNN), and a transformer-based architecture. Reported metrics include RMSE, MAE, and R2 for both training and test sets. Variants of the FCNN included adjustments to regularization, architecture depth, and optimizer settings. All models were trained using the same 140-domain dataset. Supplementary Table S5. Performance metrics (RMSE, MAE, R2, Pearson r) for both the training and test sets of the general VEFill model, evaluated across multiple feature set configurations. Each row represents a unique feature combination used to evaluate model performance. Supplementary Table S6. Domain-wise evaluation results for the general model under a LOPO strategy. The table reports RMSE, MAE, coefficient of determination (R2), and Pearson correlation (r) for both the training and test sets across 140 individual protein domains. Additionally, the number of mutations in the training and test sets for each domain is provided. Supplementary Table S7. Per-protein model performance metrics (RMSE, MAE, R2, Pearson r) for both training and test sets for 140 domains using an 90/10 random train/test split. Models were trained using all input features except ESM-1v difference vector. The table also includes the number of mut [file media-1.zip › Supplementary_Fig_S7.png]

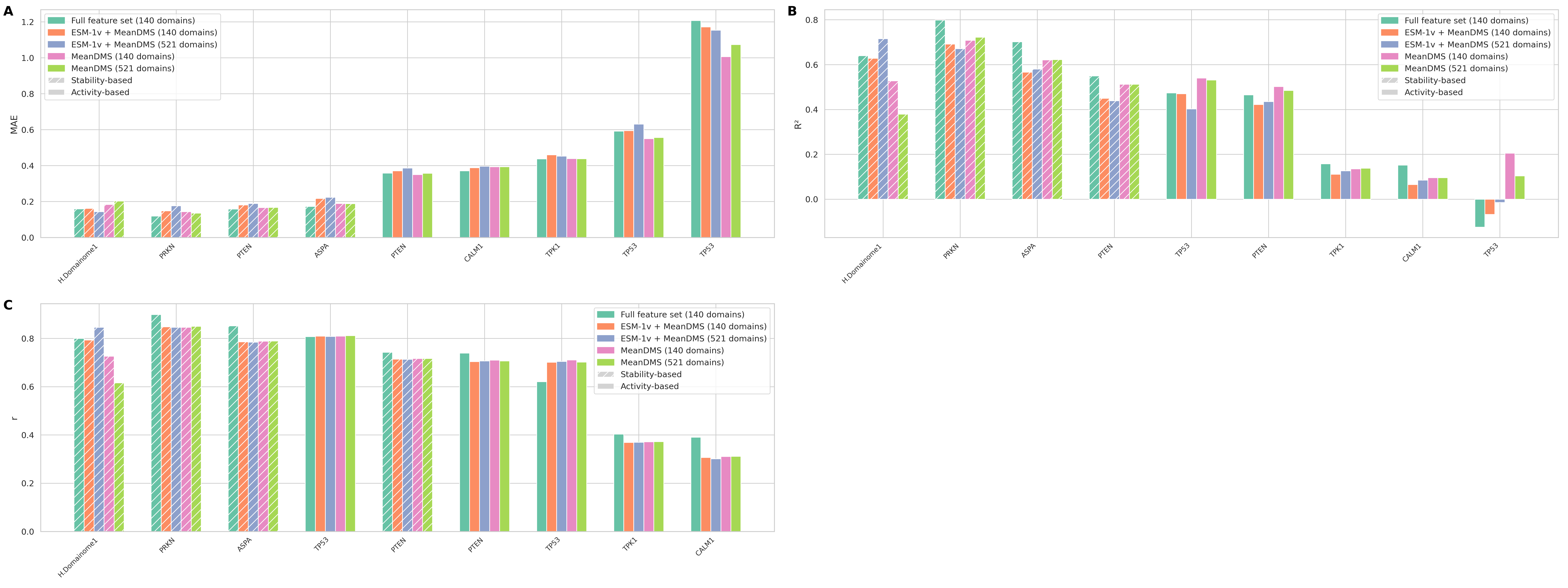

Supplement: Supplement 1 — Supplementary Table S1. Metadata for domains used in the 140-, 521-, and 127-domain (Pfam-unique) model datasets. Includes MaveDB accession, domain name, species, UniProt ID, Pfam ID. Train/test split assignments are indicated for each dataset where applicable. Supplementary Table S2. List of the 25 amino acid substitution matrices used in VEFill. Each row includes the matrix name, its source (Biopython or external repository), the corresponding VEFill feature name, and the original citation. Supplementary Table S3. Complete list of input features used in the VEFill model. Supplementary Table S4. Performance comparison of different model architectures using the same input feature set. Models evaluated include LightGBM (used in VEFill), a fully connected neural network (FCNN), and a transformer-based architecture. Reported metrics include RMSE, MAE, and R2 for both training and test sets. Variants of the FCNN included adjustments to regularization, architecture depth, and optimizer settings. All models were trained using the same 140-domain dataset. Supplementary Table S5. Performance metrics (RMSE, MAE, R2, Pearson r) for both the training and test sets of the general VEFill model, evaluated across multiple feature set configurations. Each row represents a unique feature combination used to evaluate model performance. Supplementary Table S6. Domain-wise evaluation results for the general model under a LOPO strategy. The table reports RMSE, MAE, coefficient of determination (R2), and Pearson correlation (r) for both the training and test sets across 140 individual protein domains. Additionally, the number of mutations in the training and test sets for each domain is provided. Supplementary Table S7. Per-protein model performance metrics (RMSE, MAE, R2, Pearson r) for both training and test sets for 140 domains using an 90/10 random train/test split. Models were trained using all input features except ESM-1v difference vector. The table also includes the number of mut [file media-1.zip › Supplementary_Fig_S8.png]

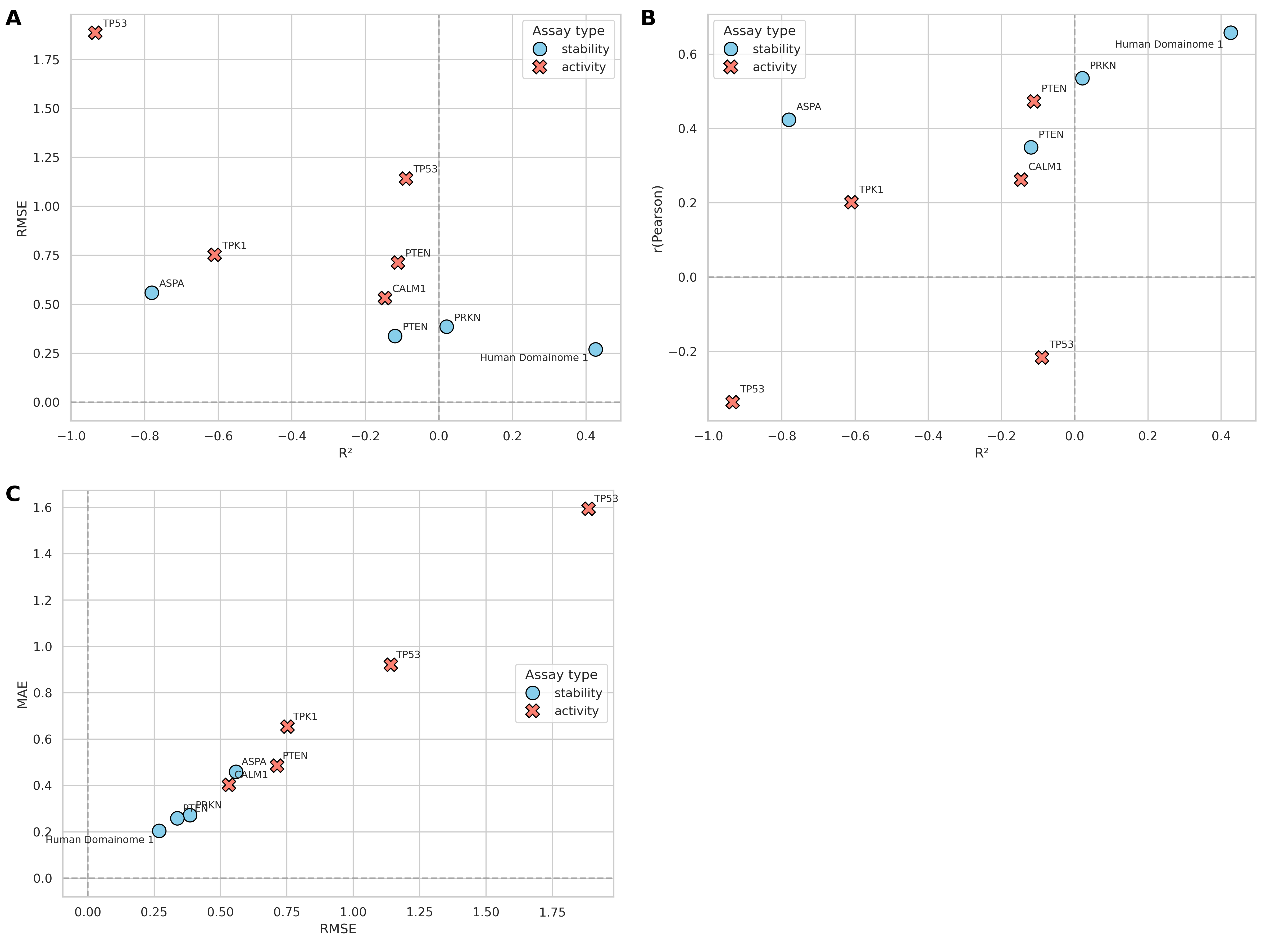

Supplement: Supplement 1 — Supplementary Table S1. Metadata for domains used in the 140-, 521-, and 127-domain (Pfam-unique) model datasets. Includes MaveDB accession, domain name, species, UniProt ID, Pfam ID. Train/test split assignments are indicated for each dataset where applicable. Supplementary Table S2. List of the 25 amino acid substitution matrices used in VEFill. Each row includes the matrix name, its source (Biopython or external repository), the corresponding VEFill feature name, and the original citation. Supplementary Table S3. Complete list of input features used in the VEFill model. Supplementary Table S4. Performance comparison of different model architectures using the same input feature set. Models evaluated include LightGBM (used in VEFill), a fully connected neural network (FCNN), and a transformer-based architecture. Reported metrics include RMSE, MAE, and R2 for both training and test sets. Variants of the FCNN included adjustments to regularization, architecture depth, and optimizer settings. All models were trained using the same 140-domain dataset. Supplementary Table S5. Performance metrics (RMSE, MAE, R2, Pearson r) for both the training and test sets of the general VEFill model, evaluated across multiple feature set configurations. Each row represents a unique feature combination used to evaluate model performance. Supplementary Table S6. Domain-wise evaluation results for the general model under a LOPO strategy. The table reports RMSE, MAE, coefficient of determination (R2), and Pearson correlation (r) for both the training and test sets across 140 individual protein domains. Additionally, the number of mutations in the training and test sets for each domain is provided. Supplementary Table S7. Per-protein model performance metrics (RMSE, MAE, R2, Pearson r) for both training and test sets for 140 domains using an 90/10 random train/test split. Models were trained using all input features except ESM-1v difference vector. The table also includes the number of mut [file media-1.zip › Supplementary_Fig_S9.png]

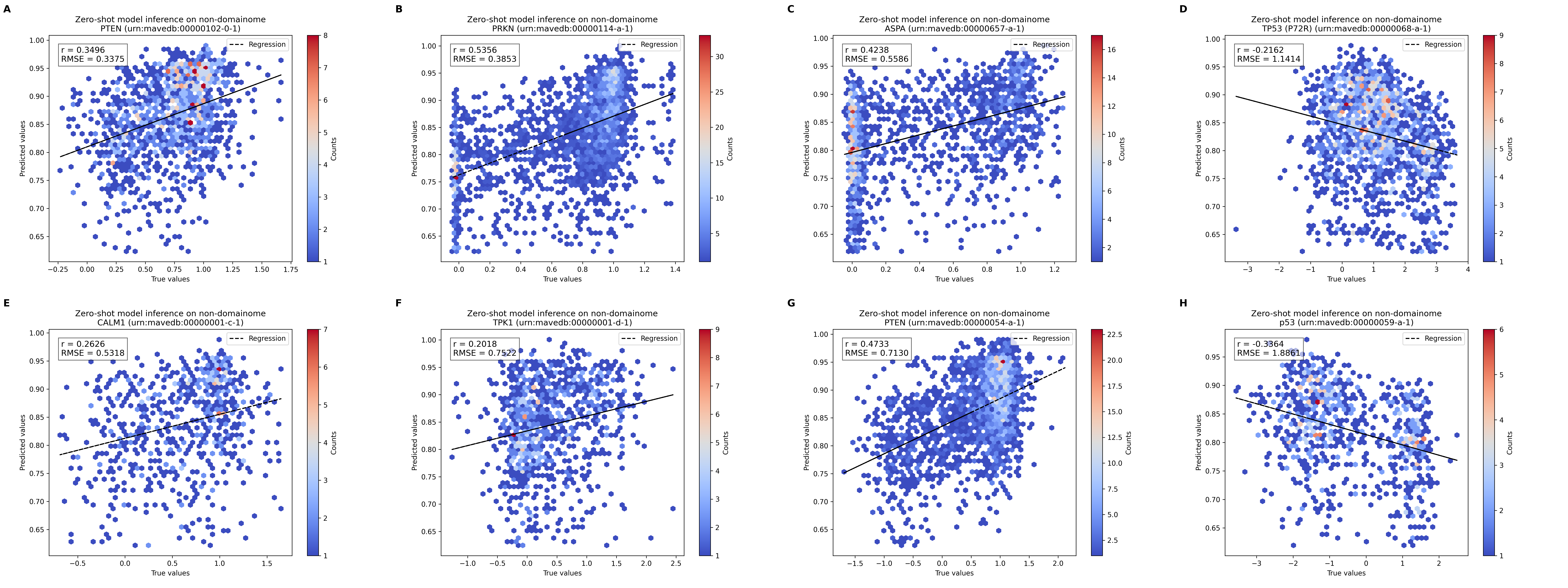

Supplement: Supplement 1 — Supplementary Table S1. Metadata for domains used in the 140-, 521-, and 127-domain (Pfam-unique) model datasets. Includes MaveDB accession, domain name, species, UniProt ID, Pfam ID. Train/test split assignments are indicated for each dataset where applicable. Supplementary Table S2. List of the 25 amino acid substitution matrices used in VEFill. Each row includes the matrix name, its source (Biopython or external repository), the corresponding VEFill feature name, and the original citation. Supplementary Table S3. Complete list of input features used in the VEFill model. Supplementary Table S4. Performance comparison of different model architectures using the same input feature set. Models evaluated include LightGBM (used in VEFill), a fully connected neural network (FCNN), and a transformer-based architecture. Reported metrics include RMSE, MAE, and R2 for both training and test sets. Variants of the FCNN included adjustments to regularization, architecture depth, and optimizer settings. All models were trained using the same 140-domain dataset. Supplementary Table S5. Performance metrics (RMSE, MAE, R2, Pearson r) for both the training and test sets of the general VEFill model, evaluated across multiple feature set configurations. Each row represents a unique feature combination used to evaluate model performance. Supplementary Table S6. Domain-wise evaluation results for the general model under a LOPO strategy. The table reports RMSE, MAE, coefficient of determination (R2), and Pearson correlation (r) for both the training and test sets across 140 individual protein domains. Additionally, the number of mutations in the training and test sets for each domain is provided. Supplementary Table S7. Per-protein model performance metrics (RMSE, MAE, R2, Pearson r) for both training and test sets for 140 domains using an 90/10 random train/test split. Models were trained using all input features except ESM-1v difference vector. The table also includes the number of mut [file media-1.zip › Supplementary_Fig_S10.png]
